# Hepatitis B Vaccine Response in Children of Vaccinated Versus Unvaccinated Mothers: A Retrospective Cohort Study

**DOI:** 10.1111/jvh.70165

**Published:** 2026-03-10

**Authors:** Safa Shibli, Mohamad Suki, Tannous Korzom, Manfred S. Green, Rifaat Safadi

**Affiliations:** ^1^ The Liver Institute, Hadassah Medical Organization, Hadassah Medical School The Hebrew University Jerusalem Israel; ^2^ School of Public Health University of Haifa Haifa Israel; ^3^ Clalit Health Services Tel Aviv Israel

**Keywords:** antibody level, antibody titre, HBV, HBV vaccine, hepatitis B virus, serological test, vaccine response

## Abstract

Universal infant hepatitis B (HBV) vaccination is highly effective in preventing infection. However, little is known about whether a mother's HBV vaccination history influences her child's immune response to routine immunisation. We aimed to compare vaccine‐induced antibody responses in children of vaccinated and unvaccinated mothers. We conducted a historical‐retrospective cohort study of 364 children who completed the standard infant HBV vaccination series and underwent post‐vaccination antibody testing. Maternal vaccination status was determined by serology, medical records and cohort year. Children were classified as offspring of HBsAg‐negative vaccinated mothers (*n* = 92), HBsAg‐negative unvaccinated mothers (*n* = 174), or HBsAg‐positive mothers (*n* = 98), who also received hepatitis B immunoglobulin (HBIG) at birth. Anti‐HBs titers were analysed and categorised. Ordinal logistic regression was used to assess associations. Overall seroprotection rates (≥ 10 mIU/mL) were high (89.3%) and did not differ significantly between groups. However, children born to mothers vaccinated prior to pregnancy were significantly more likely to achieve very high antibody levels (≥ 1000 mIU/mL) compared with children of unvaccinated mothers or those who received HBIG at birth (*p* < 0.05). This association was most pronounced in children tested at ≤ 3 years of age. Mean anti‐HBs titers were also highest in offspring of vaccinated mothers. While older maternal age and lower haemoglobin levels impaired the response, the HBIG did nothing. While infant HBV seroprotection rates were uniformly high regardless of maternal background, children born to mothers vaccinated against HBV prior to pregnancy exhibited a more robust early antibody response (≥ 1000 mIU/mL). These findings suggest a possible intergenerational enhancement of vaccine responsiveness and may affect long‐term implications for HBV vaccination strategies.

## 1 Background

Hepatitis B virus (HBV) infection remains a major global health concern, with chronic infection affecting more than 250 million people worldwide and contributing substantially to the burden of cirrhosis and hepatocellular carcinoma [1, 2]. Universal infant HBV immunisation has been highly effective in reducing perinatal and horizontal transmission, and most children (~95%) achieve protective antibody levels after completing the primary series [2, 3]. Studies indicate that anti‐HBs levels ≥ 10 mIU/mL measured 1–3 months after completing the three‐dose vaccination schedule serve as a reliable marker for vaccine‐induced protective immunity [4, 5, 6, 7]. Administering HBV vaccines alongside immunoglobulin within 12 h of birth has shown over 85% efficacy in preventing infection in infants born to HBsAg‐positive mothers [8]. Sustained immunisation programs have led to significant reductions in HBV prevalence [9, 10, 11]. This decline has been associated with reduced hepatocellular carcinoma incidence, particularly in countries that adopted early vaccination protocols [12, 13, 14].

Despite the proven efficacy of infant vaccination, variability in vaccine response exists, influenced by host genetics, environmental exposures and maternal factors. Vaccine‐induced protective antibodies (≥ 10 mIU/mL) tend to decrease over time; anti‐HBs titers were highest in children 1–3 years post‐vaccination [15, 16, 17, 18]. There is limited data about the impact of a mother's prior HBV vaccination on her child's serological outcome after routine immunisation. Some studies suggest that maternal anti‐HBs antibodies, transferred passively through the placenta, may impair infants' immune response to the HBV vaccine [19, 20]. With the expansion of universal vaccination programs, an increasing proportion of new mothers are themselves vaccine‐primed rather than naturally exposed. This shift raises important immunological and public health questions: could maternal vaccination history influence the child's immune response? Might passive transfer of maternal antibodies enhance or interfere with infant seroprotection? Understanding these intergenerational effects is crucial for informing long‐term vaccination strategies, particularly in regions with high HBV endemicity. The present study addresses this knowledge gap by comparing vaccine‐induced antibody responses in children of vaccinated mothers, unvaccinated mothers and mothers with chronic HBV infection who received hepatitis B immunoglobulin (HBIG) at birth. We hypothesized that children of vaccinated mothers would demonstrate different patterns of antibody response.

## 2 Patients and Method

### 2.1 Study Design and Population

This single‐centre retrospective study analysed data from children tested between July 2004 and September 2024, and included 266 children born to HBsAg‐negative mothers. According to Israeli Ministry of Health policy, the vaccine used throughout the study period was the recombinant HBV vaccine (Engerix‐B, GlaxoSmithKline), as per national immunisation protocols in Israel. Brand‐level details were not retrievable. Of these, 174 were children of unvaccinated mothers, and 92 were children of vaccinated mothers. The children were tested for anti‐HBs antibodies between the ages of 7 and 120 months, with corresponding maternal and child data available for each pair.

Maternal anti‐HBs data were available for only 40 out of 92 mothers (43.5%), which was insufficient for multivariable logistic regression analysis. To assess the role of passive maternal anti‐HBs antibodies, we included an additional group of 98 children born to HBsAg‐positive mothers. These children received standardised double vaccination (active and passive) at birth (HepB+HBIG). This control group consisted exclusively of children whose mothers were HBV treatment‐naïve and HepB+HBIG had no serological evidence of HBV exposure (undetectable serum HBsAg and anti‐HBc antibodies).

The cohort year study included mothers born before and after the implementation of the HBV vaccine in 1992. Mothers were categorised as vaccinated mothers (serological or documented evidence), unvaccinated mothers (no record or serology consistent with non‐vaccination) and HBsAg‐positive mothers, whose children received both HBV vaccine and hepatitis B immunoglobulin (HBIG) at birth.

Retrospective analysis was particularly important for accounting for the child's age at testing and the mother's age at delivery. Clinical and laboratory data, including anti‐HBs titers, were collected and analysed for all 364 children and their mothers.

### 2.2 Data Collection

Maternal data included age at the time of HBV testing, age at delivery and laboratory parameters such as alanine aminotransferase (ALT), aspartate aminotransferase (AST), leukocyte (WBC) count, absolute neutrophil and lymphocyte counts, haemoglobin levels, platelet count and calculated Fib‐4 scores near delivery. Children's laboratory data included WBC count, absolute neutrophil and lymphocyte counts, haemoglobin levels and platelet counts near the time of anti‐HBs testing.

Anti‐HBs titers treated as continuous variables, except for those recorded as ≥ 1000 mIU/mL, which were converted to 1001 mIU/mL to allow for accurate calculations of averages and standard deviations. Vaccine responses were then categorised into the following anti‐HBs levels: < 10 vs. ≥ 10 mIU/mL (vaccine failure vs. response), 10–999.99 mIU/mL (low response) and ≥ 1000 mIU/mL (high response). Titers ≥ 10 mIU/mL were considered protective, as per international guidelines. Titers ≥ 1000 mIU/mL were evaluated to explore associations with robust or enhanced serological responses.

### 2.3 Data Processing and Ethics

Data electronically retrieved from the Hadassah eTeams system using serological parameters (HBsAg, anti‐HBs and anti‐HBc antibodies), mother–child matching records, test dates, birth dates and child gender. Additional data, such as complete blood counts and liver enzyme levels, were extracted for mothers at delivery and children near the time of anti‐HBs testing. Missing data were manually reviewed from Hadassah's scanned medical records and laboratory reports. Identifying information was anonymized and converted into internal codes for confidentiality before statistical analysis. The study protocol was approved by the Ethics Committee at Hadassah on 23 October 2020, and remains valid until 21 October 2025. Institutional Review Board (IRB) approval was obtained (application number: 0724‐21‐HMO), along with approval from the Ministry of Health (application number: 202433983). A waiver for informed consent was granted due to the retrospective nature of the study and the anonymization of data.

### 2.4 Statistical Analysis

Baseline characteristics were summarised using medians and interquartile ranges for continuous variables and frequencies with percentages for categorical variables.

The primary outcome was the distribution of post‐vaccination anti‐HBs titers. For interpretability and comparability with prior literature, we classified antibody levels into three categories: anti‐HBs levels: < 10 mIU/mL (vaccine failure), 10–999.99 mIU/mL (low response) and ≥ 1000 mIU/mL (high response).

To evaluate associations between maternal vaccination status and antibody responses, we used ordinal logistic regression with the three antibody categories as the outcome. Models were adjusted for age at testing, sex and ethnicity. For sensitivity, multinomial regression was also performed. Multiple comparisons were controlled using false discovery rate (FDR) correction. Children with titers ≥ 1000 mIU/mL were analysed as a censored category rather than assigned arbitrary numeric values.

## 3 Results

### 3.1 Demographic and Clinical Characteristics

Baseline demographic characteristics were broadly similar across groups, although children in the HBIG group tested at a younger median age. Detailed baseline features are summarised in Table 1 that presents a one‐way ANOVA analysis of the demographic data for 364 paired mother–child groups across three study cohorts. The offspring of unvaccinated mothers group include 174 children born to HBsAg‐negative, unvaccinated mothers. The offspring of vaccinated mothers' group (reference group) with 92 children born to HBsAg‐negative, vaccinated mothers. A dual‐vaccination HepB+HBIG group having 98 children born to treatment‐naïve, HBsAg‐positive mothers who received both active and passive vaccination at birth and had no serological evidence of HBV exposure (undetected HBsAg and anti‐HBc‐Total antibodies).

**TABLE 1:** Baseline demographic and laboratory characteristics of children and mothers according to maternal HBV status.

	Overall *N* = 364	Group A	Group B	Group C	*p* ^b^	*p*		2 vs. 3
		*N* = 92^a^	*N* = 174^a^	*N* = 98^a^		1 vs. 2	1 vs. 3	
Children								
Age at test, months	26.2 ± 23.5	30.3 ± 23.2	28.7 ± 27.8	17.8 ± 8.6	< 0.001	0.6	< 0.001	< 0.001
Sex (female) N (%)	167 (45.9%)	39 (42.4%)	80 (46.0%)	48 (49.0%)	0.662			
HGB, GR%	10.9 ± 1.9	10.7 ± 1.7	11.2 ± 2.1	NA	0.138			
WBC, 10E9/L	10.5 ± 8.2	10.2 ± 7.9	10.9 ± 8.7	NA	0.582			
PLT, 10E9/L	318.7 ± 178.5	306.9 ± 168.6	332.4 ± 189.6	NA	0.385			
Mothers								
Age at HBsAg test, years	27.9 ± 11.7	22.2 ± 14.8	31.0 ± 11.7	28.4 ± 6.0	< 0.001	< 0.001	< 0.001	0.085
Age at delivery, years	28.1 ± 5.7	24.5 ± 3.8	29.9 ± 5.4	28.2 ± 5.9	< 0.001	< 0.001	< 0.001	0.008
Age at child test, years	30.2 ± 5.8	27.0 ± 4.0	32.3 ± 5.7	29.7 ± 6.1	< 0.001	< 0.001	< 0.001	< 0.001
HGB, GR%	11.8 ± 1.3	11.8 ± 1.3	11.8 ± 1.3	11.9 ± 1.4	0.909	> 0.9	0.7	0.8
WBC, 10E9/L	9.1 ± 2.7	10.0 ± 2.8	9.8 ± 1.9	7.9 ± 2.7	< 0.001	0.7	< 0.001	< 0.001
PLT, 10E9/L	214.8 ± 66.2	216.2 ± 72.1	207.4 ± 56.3	218.7 ± 67.2	0.570	0.4	0.8	0.3
ALT, U/L	20.1 ± 14.5	16.3 ± 7.8	16.8 ± 11.1	22.6 ± 17.0	0.040	> 0.9	0.017	0.2
AST, U/L	22.1 ± 9.7	19.4 ± 5.6	19.2 ± 11.1	23.9 ± 10.7	0.032	> 0.9	0.018	0.10
Fib‐4	0.7 ± 0.4	0.6 ± 0.2	0.4 ± 0.4	0.8 ± 0.4	0.002	0.090	0.024	0.002

*Note:* Baseline demographic and laboratory characteristics of 364 paired children and mothers included in the study, stratified by maternal HBV status: children born to mothers vaccinated against HBV prior to pregnancy (Group A, *n* = 92), children of unvaccinated mothers (Group B, *n* = 174) and children born to HBsAg‐positive mothers who received hepatitis B immunoglobulin (HBIG) at birth and were not exposed to HBV (Group C, *n* = 98). Data are presented as mean ± standard deviation or number (%), as appropriate. Group comparisons were performed using one‐way analysis of variance (ANOVA). Laboratory parameters include haemoglobin (HGB), white blood cell count (WBC), platelet count (PLT), alanine aminotransferase (ALT), aspartate aminotransferase (AST) and Fib‐4 index in mothers.

^a^ Mean (SD).

^b^ One‐way ANOVA.

The average children age at anti‐HBs testing was significantly younger in the HepB+HBIG group compared to the children of vaccinated and unvaccinated mothers, whereas the latter two groups had similar age distributions. Blood count parameters among the children were comparable across all three groups. Maternal age was significantly higher in the children of unvaccinated mothers' group and lowest in the children of vaccinated mothers. Notably, white blood cell (WBC) counts were significantly lower in mothers of the dual‐vaccination group (*p* < 0.001). Maternal haemoglobin levels, platelet counts and neutrophil‐to‐lymphocyte ratios showed no significant differences between groups. ALT and AST levels were higher in HBsAg‐positive mothers compared to the vaccinated group but were similar between the children of vaccinated and unvaccinated mothers. The Fib‐4 score was highest in the HepB+HBIG vaccination mothers (0.8 ± 0.4) compared to vaccinated (0.6 ± 0.2) and unvaccinated mothers (0.4 ± 0.4) groups.

### 3.2 Vaccine Response Analysis

**FIGURE 1:**
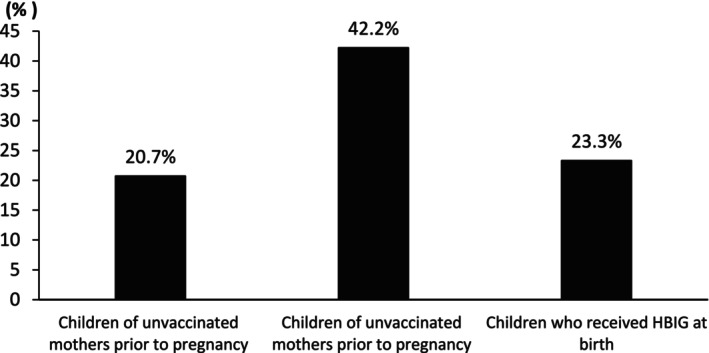
Proportion of children ≤ 3 years of age achieving very high anti‐HBs levels (≥ 1000 mIU/mL) according to maternal HBV status. Percentage of children tested at ≤ 3 years of age who achieved very high hepatitis B surface antibody (anti‐HBs) levels (≥ 1000 mIU/mL) following completion of the standard infant hepatitis B vaccination schedule. Children born to mothers vaccinated against HBV prior to pregnancy demonstrated the highest frequency (*p* < 0.05) of very high antibody responses (42.2%), compared with children of unvaccinated mothers (20.7%) and children born to HBsAg‐positive mothers who received hepatitis B immunoglobulin (HBIG) at birth (23.3%). These findings illustrate the age‐dependent enhancement of antibody responses associated with maternal pre‐pregnancy HBV vaccination.

Univariate logistic regression assessed the association of various factors with anti‐HBs response categories. The ‘children born to mothers vaccinated prior to pregnancy’ defined as the target group that was compared to the ‘children of unvaccinated mothers prior to pregnancy’ and the ‘children who received HBIG at birth and were not exposed to HBV’. Anti‐HBs responses according to maternal HBV status and age are described in Table 2 and Figure 1.

**TABLE 2:** Anti‐HBs responses according to maternal HBV status and age.

Mother status	Children	Children anti‐HBs sub‐groups (mIU/mL)				
		Mean anti‐HBs ± SD	0–9.99	≥ 10	10–999.99	≥ 1000
Unvaccinated prior to pregnancy	Total *n* = 174	392.7	24	150	116	34
		*436.5*	13.80%	86.21%	66.70%	19.50%
	Ages < 3 years *n* = 140	417.0	15	125	96	29
		*441.0*	10.70%		68.60%	20.70%
	Ages > 3 years *n* = 34	292.8	9	25	20	5
		*408.7*	26.50%	73.53%	58.80%	14.70%
Vaccinated prior to pregnancy	Total *n* = 92	521.7	10	82	50	32
		*475.9*	10.90%		54.40%	34.80%
	Ages < 3 years *n* = 64	612.1	7	57	30	27
		*475.9*	10.90%	89.06%	46.90%	42.20%
	Ages > 3 years *n* = 28	315.2	3	25	20	5
		*413.8*	10.70%	89.29%	71.40%	17.90%
HBsAg+ (*children received HepB + HBIG*)	Total *n* = 98	418.9	5	93	72	21
		*407.3*	5.10%	94.90%	73.50%	21.40%
	Ages < 3 years *n* = 90	438.3	4	86	65	21
		*410.5*	4.40%	95.56%	72.20%	23.30%
	Ages > 3 years *n* = 8	201.4	1	7	7	0
		*311.9*	12.50%	87.50%	87.50%	0.00%

*Note:* The three groups according to the status of mothers including ‘Children born to mothers vaccinated prior to pregnancy’, ‘Children of unvaccinated mothers’ and ‘children who received HBIG at birth’ were divided according to age groups (total, ≤ 3 years, > 3 years). Summary of the anti‐HBs categories (0–9.99, ≥ 10, 10–999.99 and ≥ 1000 IU/mL) shown for each age group.

Anti‐HBs responses according to maternal HBV status and age are summarised in Table 2 and Figure 1. Across the entire cohort, seroprotective anti‐HBs levels (≥ 10 mIU/mL) were achieved in 89.3% of children, with no clinically meaningful differences in overall seroprotection rates between maternal groups. Mean anti‐HBs titers showed higher average antibody levels in younger children and in offspring of mothers vaccinated against HBV prior to pregnancy; however, these estimates should be interpreted with caution, as anti‐HBs values reported as ≥ 1000 mIU/mL were truncated and assigned a value of 1001 mIU/mL for the calculation of means and standard deviations, potentially underestimating the true magnitude of high antibody responses.

Analysis by antibody response categories revealed significant differences between groups. No clinically significant differences observed anti‐HBs levels 10–999 mIU/mL (data not shown). In contrast, the proportion of children achieving very high antibody levels (anti‐HBs ≥ 1000 mIU/mL) varied by maternal status and age at testing. Compared with ‘children born to mothers vaccinated against HBV prior to pregnancy’, the odds of achieving very high anti‐HBs levels were significantly lower among ‘children of unvaccinated mothers’ (OR 0.46, 95% CI 0.26–0.81, *p* = 0.007) and among ‘children who received HBIG at birth and were not exposed to HBV’ (OR 0.51, 95% CI 0.27–0.97, *p* = 0.042).

Increasing age (per 6‐month increment; OR 0.98, 95% CI 0.97–1.00, *p* = 0.021) and higher haemoglobin levels (OR 0.90, 95% CI 0.82–0.98, *p* = 0.021) were independently associated with lower odds of achieving anti‐HBs ≥ 1000 mIU/mL. Age‐stratified analysis demonstrated that this effect was most pronounced in children tested at ≤ 3 years of age, among whom offspring of vaccinated mothers had the highest frequency of very high responses (42.2%), compared with children of unvaccinated mothers (20.7%) and children who received HBIG at birth (23.3%). In ages ≤ 3 years therefore, ‘children of unvaccinated mothers prior to pregnancy’ (OR 0.36, 95% CI 0.19–0.68, *p* = 0.002) and ‘children who received HBIG at birth and were not exposed to HBV’ (OR 0.42, 95% CI 0.21–0.83, *p* = 0.014) had lower odds of achieving this high response compared to the ‘children born to mothers vaccinated prior to pregnancy’. No significant differences were observed among children tested at > 3 years of age. No statistically differences observed in the rest children's (included gender, haemoglobin levels, white blood cell count, lymphocyte count and lymphocyte/neutrophil ratio) or mothers' variables (included gender white and platelets blood cell count, lymphocyte count, lymphocyte/neutrophil ratio, ALT & AST levels and Fib‐4 score).

Confounding factors were assessed using logistic regression analysis, with anti‐HBs levels as the dependent variable. Variables with a *p*‐value < 0.2 in univariable analysis were included in multivariate logistic regression models to evaluate the adjusted effects of independent variables while accounting for covariates. Across the entire sample and within both age subgroups at testing, increasing a child's age by 6‐month intervals was significantly associated with decreased odds of being in Anti‐HBs ≥ 1000 mIU/mL category (*p* = 0.002). No statistically differences observed in the rest selected variables included haemoglobin levels, white blood cell count, lymphocyte count and lymphocyte/neutrophil ratio.

## 4 Discussion

In this retrospective cohort study, we examined whether a mother's hepatitis B vaccination history influences her child's antibody response following routine infant immunisation. We found that overall seroprotection rates (anti‐HBs ≥ 10 mIU/mL) were similar across groups (Table 2). However, children of vaccinated mothers were more likely to develop very high antibody levels (≥ 1000 mIU/mL), particularly when tested before the age of three (Table 2; Figure 1). Our data show that the advantage conferred by maternal vaccination is most pronounced in children under 3 years of age. These findings suggest that maternal vaccination may confer an intergenerational priming effect, enhancing the robustness of vaccine‐induced immunity in early life. The significant differences in anti‐HBs titers across age groups reinforce the well‐documented decline in vaccine‐induced immunity over time [15, 16, 17]. However, it has also been extensively described that individuals who achieved anti‐HBs protective titers at the time of vaccination show a rapid anamnestic response when boosted [21, 22], suggesting memory immunity [23].

Almost all (97%) teenagers who received a booster vaccine achieved anti‐HBs levels ≥ 100 mIU/mL regardless of their pre‐booster levels [18]. For this reason, although it remains a controversial issue, a vaccine booster is not recommended currently for children and adults with normal immune status, despite the overtime drop of anti‐HBs antibody titers [23, 24]. As a booster strategy for teenagers is acceptable in some countries, our results could suggest reconsideration of such need in the children of vaccinated mothers. The lower antibody responses observed in older children underscore the potential need for booster doses, especially for children born to unvaccinated mothers. However, the booster might be of lower importance in the enhanced responses among children of vaccinated mothers. These findings may prompt further investigation into the potential role of booster vaccination in selected populations. However, as the vast majority of children achieved protective titers, any clinical implications must be interpreted cautiously and validated in prospective studies.

To understand a mechanism underlying immune deference in both vaccine generations, the role of passive maternal antibodies evaluated via the control group of ‘children who received HBIG at birth’ and were not exposed to HBV. Interestingly, the superiority of responses among children of vaccinated mothers suggests that passive maternal anti‐HBs antibodies do not suppress or boost the infant's immune response to the HBV vaccine (Table 2). This contrasts with previous studies indicating possible interference by maternal antibodies, particularly in high‐titre scenarios [19, 20].

Our observation that children of vaccinated mothers mount stronger early responses aligns with the concept of ‘trained immunity’ across generations, though the mechanisms remain speculative. Epigenetic modifications, altered maternal antibody repertoire, or in utero immune environment may play a role and warrant further study. Vaccinated mothers might passively contribute to enhanced neonatal immune readiness through factors such as cytokine milieu during gestation or epigenetic modifications in immune cells. For example, the extracellular vesicles Exosomes suggested recently having a role in HBV transmission as well as their vital roles in immune regulation during HBV infection [25]. On the other hand, placenta‐derived exosomes act as a modulator in maternal immune tolerance during pregnancy [26], suggesting a potential player in HBV vaccine immune response. These findings align with prior studies emphasising maternal contributions to neonatal immunity beyond antibody transfer [27].

Key laboratory findings provide mechanistic insights into the observed differences. Higher maternal WBC levels and neutrophil‐to‐lymphocyte ratio might reflect maternal immune readiness [28, 29], indirectly influencing foetal immune development. Moreover, there is emerging evidence that iron deficiency may limit adaptive immunity and vaccine responses [30]. In the current study, however, they did not affect odds of vaccine responses ≥ 1000 mIU/mL, underscoring the role of maternal immune status during gestation. Furthermore, maternal ALT and AST levels, alongside higher Fib‐4 scores, were not linked to vaccine response in the current study.

The clinical implications of these findings should be interpreted cautiously. The consistent tendency toward stronger responses in children born to mothers vaccinated prior to pregnancy may prove relevant for long‐term durability of immunity and for defining future vaccine strategies.

Prospective studies with larger cohorts and longitudinal follow‐up are needed to confirm these findings and explore the underlying immunological mechanisms. These findings align with growing interest in maternal immunisation strategies, not only for HBV but also for other pathogens such as influenza, pertussis and RSV, where maternal antibodies may modulate neonatal vaccine responses.

### 4.1 Limitations

This study has several limitations. First, it was a single‐centre analysis based on retrospective data from an institutional database, which may limit generalisability. Second, maternal vaccination status was verified serologically in only 40 of 92 cases. This demographic imbalance was acknowledged and adjusted in multivariate analyses.

## 5 Conclusions

Children of hepatitis B–vaccinated mothers were more likely to achieve very high antibody levels after routine infant immunisation, particularly in early childhood, while overall seroprotection remained similar across groups. These findings suggest a potential intergenerational priming effect of maternal vaccination. Prospective studies are warranted to confirm these results and clarify their implications for long‐term vaccine policy.

## Funding

The authors have nothing to report.

## Conflicts of Interest

The authors declare no conflicts of interest.

## Data Availability

The data that support the findings of this study are available from the corresponding author upon reasonable request, subject to institutional approval and ethical considerations.
